# Automaticity of Conceptual Magnitude

**DOI:** 10.1038/srep21446

**Published:** 2016-02-16

**Authors:** Yarden Gliksman, Shai Itamar, Tali Leibovich, Yonatan Melman, Avishai Henik

**Affiliations:** 1 Department of Psychology and the Zlotowski Center for Neuroscience, Ben-Gurion University of the Negev, Beer-Sheva, Israel; 2 Department of Cognitive and Brain Sciences, Ben-Gurion University of the Negev, Beer-Sheva, Israel

## Abstract

What is bigger, an elephant or a mouse? This question can be answered without seeing the two animals, since these objects elicit conceptual magnitude. How is an object’s conceptual magnitude processed? It was suggested that conceptual magnitude is automatically processed; namely, irrelevant conceptual magnitude can affect performance when comparing physical magnitudes. The current study further examined this question and aimed to expand the understanding of automaticity of conceptual magnitude. Two different objects were presented and participants were asked to decide which object was larger on the screen (physical magnitude) or in the real world (conceptual magnitude), in separate blocks. By creating congruent (the conceptually larger object was physically larger) and incongruent (the conceptually larger object was physically smaller) pairs of stimuli it was possible to examine the automatic processing of each magnitude. A significant congruity effect was found for both magnitudes. Furthermore, quartile analysis revealed that the congruity was affected similarly by processing time for both magnitudes. These results suggest that the processing of conceptual and physical magnitudes is automatic to the same extent. The results support recent theories suggested that different types of magnitude processing and representation share the same core system.

When observing an object it is clear that it has several dimensions of magnitude, some derived from intrinsic characteristics and others derived from superficial ones. Those different dimensions of magnitude are, by their nature, interconnected. It is possible that magnitude processing is a multi-dimensional process. This possibility is at the center of this study, which focuses on the interaction between conceptual (i.e., internal physical size representation of an object) and physical (i.e., perceived size of an object) magnitudes and their automaticity.

How automatic are these dimensions? Taking into account that automaticity has several definitions, we consider automaticity to be an involuntary activation of a process^1^ that is reflected in processing irrelevant information. Furthermore, automaticity is a continuum in which there are different levels, rather than an “all or none” process^2^,^3^. A common way to study automaticity is by using conflict tasks, which were first introduced by Stroop^4^,^5^. For example, participants are presented with a two-dimensional stimulus and asked to respond to one dimension and ignore the other. The effect of the irrelevant (to-be-ignored) dimension on performance is an indication of this dimension’s automaticity.

Such conflict tasks were used to ask if magnitudes are processed automatically. Paivio^6^ was the first to address the interaction between physical and conceptual magnitudes. First, participants were asked to scale the conceptual magnitude of objects on a scale of 1 to 10. Based on those norms, group of participants were indepenently presented with drawings of pairs of objects that appeared in different physical magnitudes and were asked to indicate which one was conceptually larger. This design created congruent (e.g., a physically small lamp compard with a physically large zebra) and incongruent) e.g., a physically large lamp compard to a physically small zebra) conditions. It was found that larger conceptual distances resulted in shorter reaction times (RTs), termed the distance effect (see also Moyer^7^ and Setti, Caramelli & Borghi^8^). In addition, it was found that congruity modulated RT; namely, congruent trials were significantly faster than incongruent trials. These findings were taken as evidence for the automatic processing of physical magnitude, which was processed when irrelevant to the task. Moreover, these findings were found regardless of notation (i.e., object drawings or words). Importantly, this experimental design did not allow examining the automaticity of conceptual magnitude.

The automaticity of conceptual magnitude was examined by Rubinsten and Henik^9^, who presented participants with two words of animal names and asked them to choose the larger one. In separate blocks of trials, participants were directed to choose the larger stimulus according to either the physical (e.g., which word was larger on screen) or conceptual (e.g., which word represented the larger animal) magitudes. The stimuli differed in both their physical and conceptual magnitudes, creating congruent and incongruent comparisons. RTs were faster for congruent than for incongruent trials; namely, there was size congruity effect, regardless of task (physical or conceptual). This suggests that both physical and conceptual magnitudes were processed automatically. Moreover, it was found that even though the congruity effect in the conceptual task was significantly larger than in the physical task, proportional effect analysis revealed no differences in the congruity effect between the tasks. This in turn might suggest that the physical magnitude is as automatic as the conceptual magnitude. Unlike Paivo’s study^6^ the aforementioned study used words and not object drawings to elicite conceptual magnitude. It was not not clear, however, whether conceptual magnitudes presented as object drawings were proccessed automatically.

The interaction between physical and conceptual magnitudes (using pictures of objects) was recently investigated by Konkle and Oliva^10^. In their study, participants were presented with images of familiar objects. The objects differed in both their physical and conceptual magnitudes. Objects were divided into two groups—small objects (e.g., cherry, camera, etc.) and big objects (e.g., door, train, etc.)—and appeared in two different physical sizes on the screen. Participants were asked to determine which object appeared larger on the screen (i.e., physical comparison task). The physical and conceptual sizes were either congruent or incoungruent, thus allowing for the appraisal of automaticity of conceptual magnitude. The results indicated that conceptual magnitude was processed automatically.

Nevertheless, two aspects of Konkle and Oliva’s study^10^ may limit such a conclusion. First, every incorrect response was followed by error feedback and an interval of 5 seconds before the next trial began, whereas correct responses were followed by a 900 ms interval before the next trial started. Besides creating an unbalanced design, this could have inadvertently increased the participents’ sensitivity to a conflict and swayed them to slow down whenever a conflict appeared. As a result, the congruity effect that was found might not have reflected the actual automaticity level of the conceptual magnitude. Second, the differences between the compared objects were not equated for physical and conceptual dimensions; pairing a paintbrush with a train, or a camera with a tractor reflected extreme differences in the ratio of conceptual magnitudes, whereas the stimuli’s physical sizes differed roughly in a ratio of 0.5 (i.e., smaller divided by larger size). Given what is known about the influence of ratio effect on congruity (e.g., Leibovich, Diesendruck, Rubinsten & Henik^11^), such a design might have incresased the saliency of the conceptual dimension in respect to the physical dimension, and thus have enabled the automatic processing of conceptual magnitudes.

While it is clear that both physical and conceptual magnitude is automatically processed when the stimuli are words^6^,^8^,^9^, it remains unclear whether the conceptual magnitude is automatically processed when the stimuli are drawings. It is also unclear if physical and conceptual magnitudes are automatically processed to the same extent.

## The Current Study

The aim of the current study was to examine whether conceptual magnitude conveyed by drawings is automatically processed, as was previously suggested^10^. Moreover, we aimed to explore the relationship between the levels of conceptual and physical automaticity; namely, we wanted to eamine if the processing of conceptual and physical magnitudes are automatically processed to the same extent. For that purpose, we employed a Stroop-like conflict task. Namely, participants were presented with pictures of drawn objects in different physical sizes and were asked, in separate blocks of trials, to indicate which object was larger physically or conceptually. We hypothesised that physical magnitude would be automatically processed and would thus would create a congruity effect when irrelevent to the task. In addition, we expected that the physical task would result in significantly faster RTs compared to the conceptual task. This prediction was based on findings showing that physical magnitude is highly automatic^6^,^9^,^12^,^13^. For conceptual magnitude, based on Rubinstein and Henik’s^9^ and Konkle and Oliva’s^10^ findings, we hypothesised that conceptual magnitude would also be automatically processed.

## Results

### Exclusion of data

The exclusion of data was done in several steps. First, we calculated the average ACC for each pair across participants. Pairs with ACC larger than 2 standard deviations (SD) from the average were excluded; this resulted in five pairs being excluded across subject data. Next, we calculated the average ACC for each participant. Participants whose ACCs were larger than 2 SD from the average ACC were excluded; this resulted in one participant being excluded. After this, error trials were excluded. This resulted in excluding 8.3% of the data from further analyses (1.9% and 15.2% for the physical and conceptual tasks, respectively). Finally, for each participant in each condition, RTs that were 2 SD smaller or larger than the average were excluded. This resulted in excluding 4.6% of the data from further analyses (4.3% and 5% for the physical and conceptual tasks, respectively).

### Analysis

**Figure 1:**
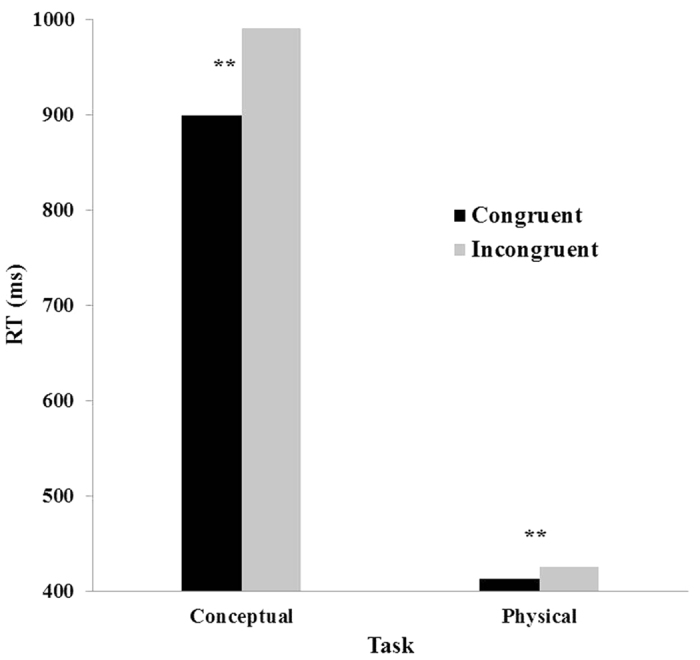
Results of the conceptual and physical tasks in Experiment 1. A significant congruity effect is marked by an asterisk. **p < 0.01.

Mean RTs were calculated for the included responses only and subjected to a two-way analysis of variance (ANOVA) with task (physical and conceptual) and congruity (congruent and incongruent) as independent variables. All main effects were significant. The physical task was faster than the conceptual task (419 ms and 945 ms, respectively), *F* (1, 18) = 203.64, *MSE* = 464,403, *p *< 0.001, *η*_*p*_^*2*^ = 0.91. Congruent trials were faster than incongruent trials (657 ms and 708 ms, respectively), *F* (1, 18) = 137.31, *MSE* = 6,739, *p *< 0.001, *η*_*p*_^*2*^ = 0.88. The task X congruity interaction was significant, *F* (1, 18) = 37.46, *MSE* = 14,292, *p *< 0.001, *η*_*p*_^*2*^ = 0.68. Contrast analyses revealed that the congruity effect was significant in the physical task, *F*(1, 18) = 10.57, *MSE* = 2,507, *p *< 0.01, *η*_*p*_^*2*^ = 0.37, as well as in the conceptual task, *F*(1, 18) = 77.42, *MSE* = 18,523, *p *< 0.001. *η*_*p*_^*2*^ = 0.81. The congruity effect was larger in the conceptual task (91.5 vs. 12.5 ms) (see Fig. 1).

**Figure 2:**
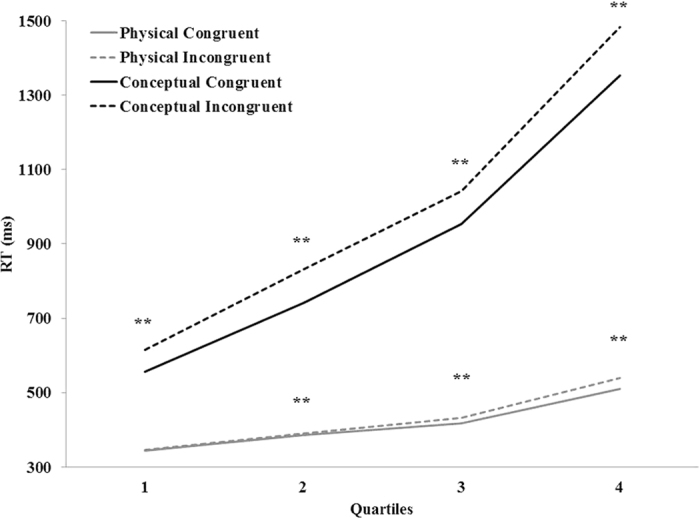
Quartiles analysis. A significant congruity effect is marked by an asterisk **p < 0.01.

Because general RTs were much faster in the physical task, we examined the role of speed of processing in modulating the congruity effect in the two tasks. Quartile analysis is widely used for the examination of processing speed on behavioral effects^14^,^15^,^16^,^17^. In this analysis RTs were divided into 4 quartiles, from the fastest to the slowest (i.e., 25%, 50%, 75%, 100%) for each participant, separately for each task. The number of trials included in the quartiles was a minimum of 21 trials per subject in each condition, and ranged between 21 to 38 trials. The data was subjected to a three-way ANOVA with task (physical and conceptual), congruity (congruent and incongruent) and quartile (25%, 50%, 75% and 100%) as independent variables (see Fig. 2).

All main effects, as well as the interaction between task and congruity were significant as in the original analysis. The interaction of quartile and congruity was significant, *F*(1, 18) = 12.92, *MSE* = 33,020, *p *< 0.001, *η*_*p*_^*2*^ = 0.41. The interaction of quartile and task was significant, *F*(1, 18) = 145.07, *MSE* = 551,863, *p *< 0.001, *η*_*p*_^*2*^ = 0.89. The interaction of task X congruity X quartile was not significant, *F*(3, 54) = 1.89, *MSE* = 46,833, *p* = 0.14, *η*_*p*_^*2*^ = 0.09. In order to reveal the modulation of speed of processing–quartile on congruity—in each task, we first examined the simple interaction and then examined the congruity effect for each quartile. In the physical task, the quartile X congruity interaction was significant, *F*(3, 54) = 5.58, *MSE* = 13,000, *p *< 0.001, *η*_*p*_^*2*^ = 0.24. In the 25% quartile the congruity effect was not significant, *F*(1, 18) = 1.44, *MSE* = 652, *p* = 0.25, *η*_*p*_^*2*^ = 0.07, whereas, in the 50%, 75% and 100% quartiles the congruity effect was significant, *F*(1, 18) = 8.64, *MSE* = 642, *p *< 0.01, *η*_*p*_^*2*^ = 0.32; *F*(1, 18) = 15.97, *MSE* = 1,896 *p *< 0.001, *η*_*p*_^*2*^ = 0.47; and *F*(1, 18) = 7.29, *MSE* = 19,778, *p* = 0.01, *η*_*p*_^*2*^ = 0.29, respectively. Furthermore, a liner trend of congruity in the different quartiles was found to be significant, *F*(1, 18) = 6.61, *MSE* = 9,952, *p *< 0.05, *η*_*p*_^*2*^ = 0.27.

In the conceptual task, the quartile X congruity interaction was also significant, *F*(3, 54) = 6.62, *MSE* = 66,853, *p *< 0.001, *η*_*p*_^*2*^ = 0.26. The congruity effect was significant in all quartiles; *F*(1, 18) = 24.58, *MSE* = 24,447, *p *< 0.001, *η*_*p*_^*2*^ = 0.57; *F*(1, 18) = 145.07, *MSE* = 9,425, *p *< 0.001, *η*_*p*_^*2*^ = 0.89; *F*(1, 18) = 44.67, *MSE* = 30,455, *p *< 0.001, *η*_*p*_^*2*^ = 0.71; and *F*(1, 18) = 38.84, *MSE* = 75,283, *p *< 0.001, *η*_*p*_^*2*^ = 0.68, for 25%, 50%, 75% and 100% quartiles, respectively. Furthermore, a liner trend of congruity in the different quartiles was found to be significant, *F*(1, 18) = 11.08, *MSE* = 35,429, *p *< 0.01, *η*_*p*_^*2*^ = 0.38. Finally, in order to examine the possible differences between the effects of quartile on congruity between tasks, we contrasted the linear trends between the tasks.

This contrast was not significant, *F*(1, 18) = 2.46, *MSE* = 27,794, *p* = 0.13, *η*_*p*_^*2*^ = 0.12. The non-significant contrast (null effect) can imply that processing time modulated the congruity effect in a similar way in both tasks. Since the null hypothesis could not be confirmed using classical statistics, we used Bayesian statistics. Bayesian statistical analyses result in decisions based on the ratio between the probability of the data given H_0_ and the probability of the data given H_1,_ namely, the Bayes Factor (BF) statistic. The BF can be calculated either as BF_10_ (meaning that the probability of the data given H_0_ is the denominator) or as BF_01_ (meaning that the probability of the data given H_1_ is the denominator). A result of BF_10_ = 10 means that the data is ten times more likely under the alternative hypothesis. Importantly, Bayesian ANOVA is similar to model selection in regression. Specifically, BF for the entire model (composed of the main effects and interactions) reflects its fit, as a whole model, to the data, where a higher value indicates a better fit of the alternative hypothesis compared to the null hypothesis. The analysis was conducted by using JASP statistical software^18^. A Bayesian repeated-measure ANOVA^19^ was conducted with task (physical and conceptual), congruity (congruent and incongruent) and quartile (25%, 50%, 75% and 100%) as independent variables. Regarding the question in hand—whether processing time modulated the congruity effect in a similar way in both tasks—we compared the BF values of two suggested models: first, a model that included all main effects and all 2-way interactions (*BF*_*10*_ = 7.018 × 10^131^ compared to the null model, which only included the participants); second, a model that included all the parameters of the first model and the 3-way interaction (*BF*_*10*_ = 5.774 × 10^130^). BF_comparison_, calculated as the ratio between the BFs of these two models, reflects by how much the former is more likely than the latter. BF_comparison_ was 12.15. Accepted norms regard BFs between 10 and 30 as “strong evidence”^20^,^21^. Accordingly, the data provided strong evidence against the 3-way interaction. Taken together, the model that fitted the data better did not include the effect of task in the interaction between quartile and congruity. This comparison of BFs of the two models supports our null hypothesis, that processing time modulated the congruity effect in a similar way regardless of task.

## Discussion

When we see a picture of a lemon, do we process the object’s conceptual magnitude automatically and to the same extent as we automatically process physical magnitude? We found that while both dimensions were processed automatically, conceptual magnitudes were processed more slowly than physical magnitudes were. Further analysis using Bayesian statistics revealed that in both dimensions, automatic processing was similar. The effect of processing speed on automaticity did not differ between magnitude dimensions.

When considering our results and those reported by Rubinsten and Henik^9^, we suggest that an object’s conceptual magnitude, whether the object is presented as a word or a drawing, is processed automatically. Furthermore, the level of automaticity of conceptual magnitude is similar to that of physical magnitude. This similarity in automaticity is not the only aspect that both physical and conceptual magnitude share; since Rubinsten and Henik^9^ found conceptual automatic processing when presenting as name words, it is clear that conceptual magnitude is automatically processed regardless of notation, just as physical magnitude is^6^.

Considering that conceptual magnitude is an internal physical size representation of an object, supported by our results, it is clear that conceptual magnitude is fundamentally a continuous magnitude. Recent theories suggested that different types of magnitude (i.e., symbolic, discrete, continuous magnitudes) processing and representations share the same core system^22^,^23^,^24^. Therefore, it is not surprising that the automatic processing of continuous magnitudes is not essentially different. However, participants responded significantly faster in the physical task than in the conceptual task. This pattern was also found when conceptual notation was name words^9^. The discrepancy between processing time of physical and conceptual magnitudes can be due to different cortical processing pathways. Many reports suggested the involvement of the intraparietal sulcus (IPS) in magnitude processing^25^,^26^,^27^. Namely, it was suggested in both primates and humans studies that cell populations in the IPS respond to magnitude information (regardless of magnitude dimension) in comparative judgment and discrimination tasks^25^. It is in the IPS that the automatic processing of either conceptual or physical magnitudes accrues, and in the same manner. However, the extraction of conceptual magnitude information requires the identification and processing of the given object. This process involves the ventral temporal cortex (also known as the ventral “what” stream) in which objects are recognized^28^,^29^,^30^. Taken together, it is possible that the processing time differences between physical and conceptual magnitudes is due to the fact that in order to process conceptual magnitude in the IPS, the object first needs to be recognized in the ventral temporal cortex. Importantly, in spite of the difference in processing requirements of the two types of magnitude (conceptual and physical), both are carried out automatically. This theoretical suggestion should be further studied.

To further support our findings and conclusions, future studies should incorporate measures for speed of processing. In this way, it will be possible to examine ad hoc the role of speed of processing in automatic processing.

To conclude, our study addressed the issue of conceptual magnitude automaticity processing. It is important to expand our knowledge on the processing of conceptual magnitude. Because conceptual magnitudes are acquired earlier than symbolic magnitudes (before school age), this primacy of conceptual magnitude can open up new ways to study normal and abnormal developmental trajectories of numerical cognition.

## Methods

### Participants

Twenty participants (10 females, mean age 24.75 years old) from Ben-Gurion University of the Negev participated in the experiment for money (about $6 per hour). All were native Hebrew speakers, with normal or corrected-to-normal vision and without any reported learning disabilities. Informed consent was obtained from all subjects. This study was approved by the psychology department’s ethics committee at Ben-Gurion University of the Negev.

### Stimuli

**Figure 3:**
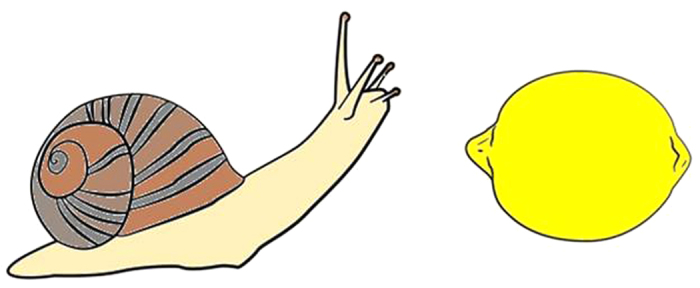
An example of an incongruent trial—the snail is physically larger than the lemon. Note, the image is a representation of the stimulus. The stimuli in the experiment were taken from Rossion and Pourtois’^31^ image set.

The experiment was programmed in E-Prime 2.0. Stimuli consisted of two familiar objects, taken from Rossion and Pourtois^31^ image set. Each object had two dimensions of magnitude: physical and conceptual. This allowed creating congruent (the conceptually larger object was physically larger) and incongruent (the conceptually larger object was physically smaller) pairs of objects. The pairing of objects was based on a conceptual magnitude index. In this index an independent group of subjects rated 60 objects and gave each a numerical value. This allowed calculating the conceptual ratio (small/large) between all pairs (for more details see supplementary materials). The criterion to select object pairs was that conceptual ratio between the objects was 0.45–0.55. This resulted in selection of 40 different objects that created 58 pairs (see Table 1). The stimuli appeared in color against a white background. Each object appeared on one side of the screen midpoint, as illustrated in Fig. 3. The center-to-center distance between the two objects was 10 cm. Participants viewed the stimuli from a distance of approximately 60 cm, creating a viewing angle of 3.5°.

### Design

Participants performed the physical and conceptual tasks in separate blocks. The order of the tasks was counterbalanced across participants. Congruity was manipulated orthogonally for each task. The ratio between the objects’ magnitude, both physical and conceptual, was between 0.45 and 0.55 (smaller size/larger size). Small to large sized objects (e.g., snail, lemon, fox, fridge and rhino, respectively) appeared in small to large physical sizes (in pixels: 82.5 × 165, 110 × 220, 127.5 × 255, 145 × 290 and 162.5 × 325). Each participant preformed 640 trials; task (2) X congruity (2) X object side (2) X repetition (80; random sampling out of 58 pairs). Each task began with 10 practice trials followed by 4 blocks of 80 trials, with a break between blocks. Dependent variables were RT, recorded in milliseconds (ms), and accuracy (ACC).

### Procedure

**Figure 4:**
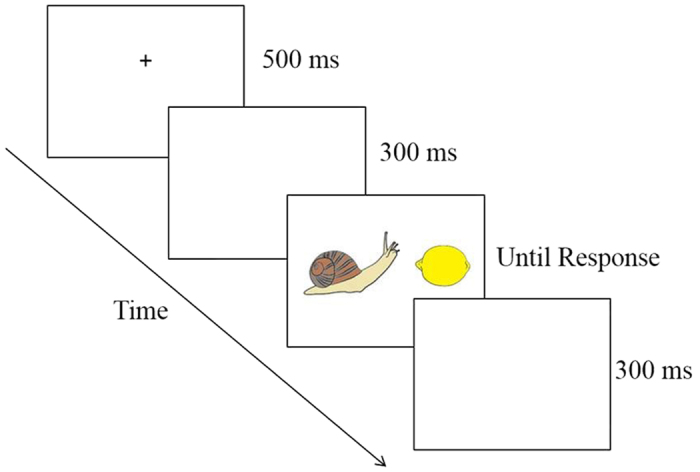
An example of an experimental trial sequence. Note, the image is a representation of the stimulus. The stimuli in the experiment were taken from Rossion and Pourtois’^31^ image set.

The following procedure was carried out in accordance with the psychology department’s ethics committee at Ben-Gurion University of the Negev guidelines. Participants carried out the experiment in the laboratory, in a sound-attenuated, dimly lit room. In separate blocks of trials, they were asked to indicate which object (left or right) was larger physically/conceptually. Responding was made by key presses, using the Q key for the left side response and the P key for the right side response. Participants were instructed to respond as quickly and as accurately as possible. Each trial began with a central fixation cross, presented for 500 ms. Three hundred ms after the elimination of the fixation cross, the stimulus appeared and remained in view until the participant pressed a key. The next trial started 300 ms after response onset (see Fig. 4).

## Additional Information

**How to cite this article**: Gliksman, Y. *et al.* Automaticity of Conceptual Magnitude. *Sci. Rep.* **6**, 21446; doi: 10.1038/srep21446 (2016).

## Supplementary Material

Supplementary Information

## Figures and Tables

**Table 1:** Pairs used in the experiment.

Bear	Zebra	Hat	Frog
Bear	Leopard	Horse	Bed
Bed	Cart	Horse	Fridge
Book	Pear	Horse	Zebra
Book	Lemon	Lemon	Snail
Book	Onion	Lemon	Butterfly
Book	Cup	Leopard	Table
Book	Glass	Leopard	Oven
Butterfly	Bee	Leopard	Desk
Butterfly	Button	Leopard	Door
Cart	Garbage	Leopard	Dresser
Chicken	Grapes	Motorcycle	Bed
Cow	Couch	Motorcycle	Fridge
Cup	Butterfly	Onion	Snail
Desk	Cask	Onion	Butterfly
Desk	Fox	Oven	Cask
Donkey	Table	Oven	Fox
Donkey	Oven	Pear	Snail
Donkey	Desk	Pan	Bird
Donkey	Door	Pan	Fish
Donkey	Dresser	Rhino	Cow
Door	Cask	Table	Cask
Door	Fox	Table	Fox
Dresser	Cask	Teapot	Fish
Dresser	Fox	Teapot	Grapes
Fridge	Cart	Toaster	Book
Glass	Butterfly	Turtle	Fish
Hat	Cup	Zebra	Cart
Hat	Glass	Zebra	Table
