# Supplementary material for: Automaticity of Conceptual Magnitude
Source: Sci Rep. 2016 Feb 16;6:21446. doi: 10.1038/srep21446 (PMC4754897; doi:10.1038/srep21446)
Supplement: Supplementary Information [file srep21446-s1.pdf]

Automaticity of Conceptual Magnitude

Yarden Gliksman[1*+] Shai Itamar[1*+], Tali Leibovich[2], Yonatan Melman[1] and Avishai Henik[1, 2]

[1]Department of Psychology and the Zlotowski Center for Neuroscience, Ben-Gurion University of the Negev, Beer-Sheva, Israel

[2]Department of Cognitive and Brain Sciences, Ben-Gurion University of the Negev, Beer-Sheva, Israel

Supplementary Materials: Conceptual Magnitude Ranking

Objectives

In order to compare objects according to their conceptual magnitude a conceptual scale is needed. Since conceptual magnitude cannot be objectively measured, as opposed to physical magnitude, there is a need for a subjective measurement. A common method for acquiring subjective measurements is number ranking using references [1,2]. To do this, we conducted two experiments. The first experiment was conducted in order to examine whether objects are recognizable and at what speed of presentation. Furthermore, we examined if recognition of objects would change as a result of presenting them in different physical sizes. The second experiment was conducted in order to establish a conceptual magnitude scale of different objects.

Experiment 1: Object Recognition

Method

Participants

Thirty participants (25 females, mean age 23.16 years old) from Ben-Gurion University of the Negev participated in the experiment for money (about $6 per hour). All were native Hebrew speakers, with normal or corrected-to-normal vision and without any reported learning disabilities. This study was approved by the psychology department's ethics committee at Ben-Gurion University of the Negev.

Object set

The object set consisted of 58 colored drawn objects, taken from Rossion and Pourtois' [3] image set. Objects were selected to keep a general, uniform-shaped object set (e.g., not elongated, perforated or segmented objects).

Stimuli

The experiment was programmed in E-Prime 2.0. Stimuli consisted of single objects, taken out of the object set. The object appeared in color against a white background, as illustrated in Figure S1.

Design

Participants performed the recognition task in one block. Each object appeared in 4 different physical sizes (in pixels: 82.5 X 165, 110 X 220, 145 X 290 and 162.5 X 325), which were randomly selected within the task. Each participant preformed 240 trials (i.e., 60 objects X 4 physical sizes). Dependent variables were reaction time (RT), recorded vocally in milliseconds (ms), and accuracy (ACC).

Procedure

Participants carried out the experiment in the laboratory, in a sound-attenuated, dimly lit room. Each task began with a brief explanation given by the experimenter, which was followed by adjustment of the microphone to the participant's voice loudness. Participants were asked to name the object that was presented on screen as fast as they could. A microphone was used to register vocal input. RT was recorded electronically by a response box controlled by E-Prime software and was measured from onset of the stimulus to onset of the vocal response. Each trial began with a central fixation cross that was presented for 300 ms. This was followed by the appearance of the object at the center of the screen, which remained in view until the participant gave a vocal response. This was followed by a blank screen that remained until the experimenter indicated whether the response was correct (i.e., 1), incorrect (i.e., 0), or deviant (i.e., 2; e.g., in cases when the participant gave another vocal response, such as "amm"). This was followed by the next trial (see Figure S1).

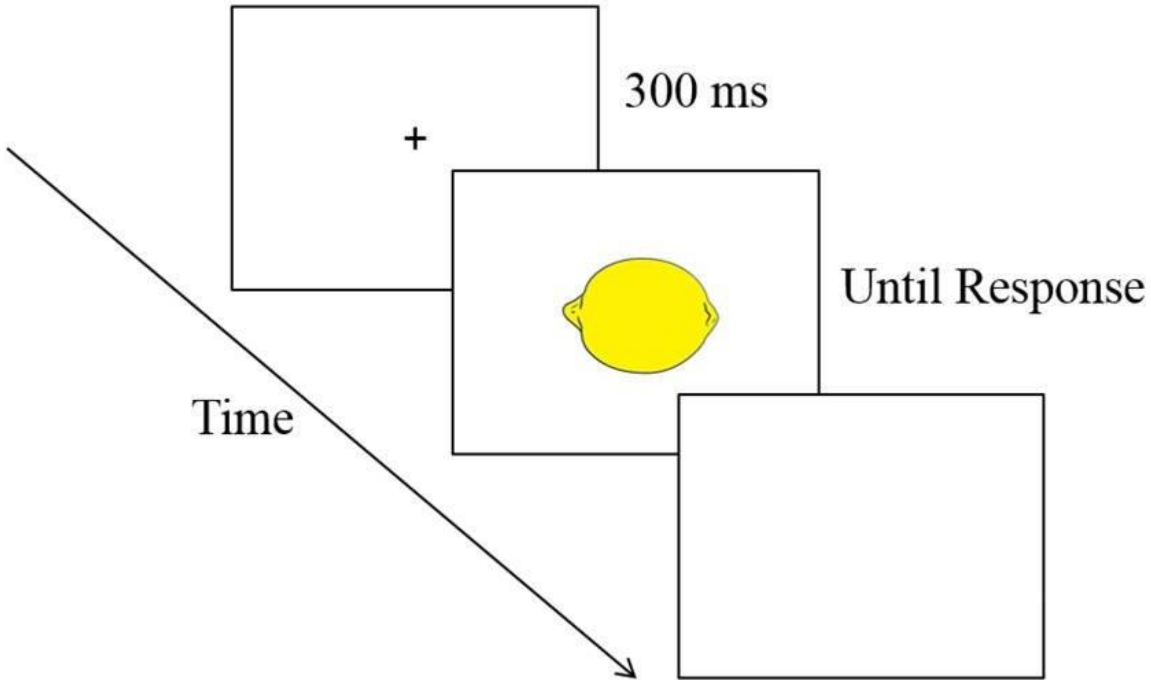

Figure S1. An example of an experimental trial. Note, the image is a representation of the stimulus. The stimuli in the experiment were taken from Rossion and Pourtois' [3] image set.

Results

Data was subjected to three analyses. First, we analyzed and excluded objects for which the mean ACC was low. Second, we analyzed and excluded objects for which the mean RT was high or low. Third, we analyzed and excluded objects for which their recognition RTs were modulated by physical size levels.

ACC analysis

Deviant responses were excluded. Mean ACC was calculated for each object, and both mean and standard deviation were calculated for the objects group; mean = .98, standard deviation = .037. A low ACC mean was defined as being lower than two

standard deviations from the ACC group mean; namely, when ACC was lower than 91%. This resulted in excluding one object from further analysis: Gorilla (mean ACC = 0.72).

RT analysis

Mean RT was calculated for each object, and both mean and standard deviation were calculated for the objects group; mean = 829 ms, standard deviation = 89.05 ms. The low/high RT mean was defined as the RT mean that was lower/higher than two standard deviations from the group mean; namely, RTs lower than 651 ms or higher than 1,007 ms. This resulted in excluding three objects from further analysis: Deer (mean RT= 1,041 ms), Sheep (mean RT = 1,045 ms), and Beatle (mean RT = 1,079 ms).

Physical sizes modulation analysis

Mean RTs for each object were subjected to a one-way analysis of variance (ANOVA) with physical size (in pixels: 82.5 X 165, 110 X 220, 145 X 290 and 162.5 X 325) as an independent variable. The details of each one of the 57 ANOVAs conducted (*F*s and *p* values can be seen in Table S1). This resulted in excluding one object from further analysis due to a significant *p*-value that was lower than .05: Tomato ($F = 5.68$, $p = 0.001$).

Experiment 2: Conceptual Magnitude Scaling

Method

Participants

Thirty participants (14 females, mean age 22.03 years old) took part following the same participant criteria and guidelines as in Experiment 1.

Object set

The object set consisted of 53 colored drawn objects, taken from the Rossion and Pourtois' [3] object set used in Experiment 1 after the exclusion of 5 objects (as described in Experiment 1 above).

Stimuli

The experiment was programmed in E-Prime 2.0. Stimuli consisted of a two objects, one was the target object (which varied across the experiment and was taken out of the object set) and the other was the reference object (i.e., chair). The objects appeared in color against a white background.

Design

Participants performed the conceptual scaling task in two blocks. Both objects (i.e., target and reference) appeared in the same physical sizes (145 X 290 pixels). The location of both target and reference objects (i.e., right or left of the screen center) was counterbalanced. Each participant preformed 212 trials (i.e., 53 objects X 2 sides X 2 repetitions. The dependent variable was size scaling of the target objects, reported as a number (1-1,000,000) by the participants.

Procedure

Participants carried out the experiment in the laboratory, in a sound-attenuated, dimly lit room. Each task began with a brief explanation given by the experimenter. Participants were asked to rate the real-world size of the target object that was presented on screen by typing a number. They were instructed to do so in reference to the reference object—a chair whose real-world size was set to 500. Each trial began with presentation of a fixation cross presented for 300 ms and then another blank screen presented for 200 ms. Then, two objects appeared (i.e., a chair and another object) and the participant had

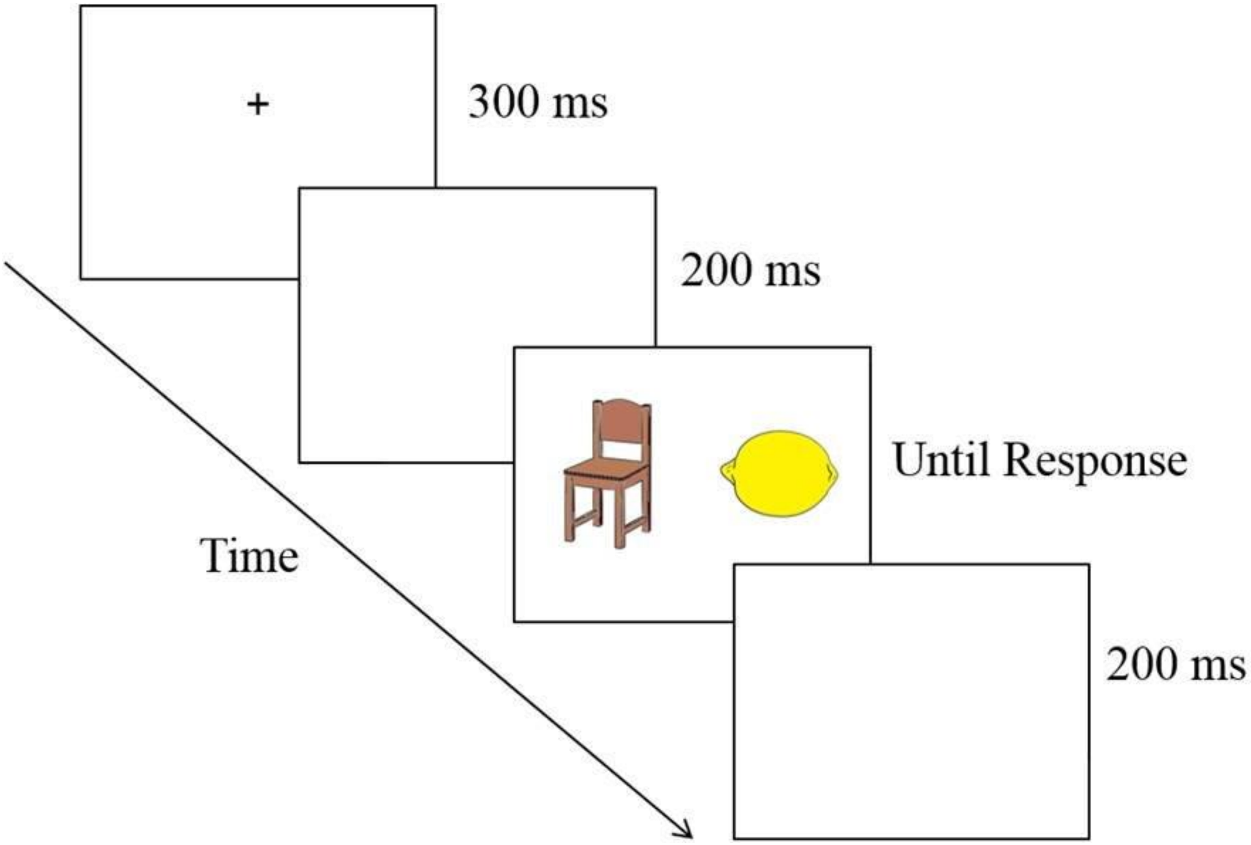

to type the size of the object. The objects remained in view until the participant gave a size rating response). This was followed by a blank white screen for 200 ms, after which the next trial began (see Figure S2).

Figure S2. An example of the experimental trail. Note, the image is only a representation of the stimulus. The stimuli were taken from Rossion and Pourtois' [3] image set.

Results

Data was subjected to two analyses. First, we analyzed and excluded participants whose ordinal ratings were weakly correlated to the ordinal scaling of a group of independent raters. Second, we analyzed and excluded objects whose size ratings were relatively unstable.

Ordinal analysis

We asked 10 independent raters to ordinally list the objects according to their real-world size. Results were used to create an average rater ordinal list (AOL). A Spearman correlation matrix was created for all the raters' lists as well as for the AOL. Two raters' results were excluded since their ordinal size rating Spearman correlation to the AOL was lower than 2 standard deviations from the general Spearman correlation (average Spearman correlation of all raters to the AOL, $p_s = .95$, $SD = .024$). This was followed by calculating each participant's mean size judgement of each object. This enabled us to create an ordinal list of the objects according to the size rating for each participant. A similar Spearman correlation matrix was created for the participants' ordinal ratings and the AOL. One participant was excluded since his ordinal size rating Spearman correlation to the AOL was lower than 2 standard deviations from the general Spearman correlation (average Spearman correlation of all participants to the AOL, $p_s = .92$, $SD = .17$).

Size rating stability analysis

Besides calculating the mean size rating for each object for each participant, we calculated the Coefficient of Variance (CV; object size standard deviation divided by its mean size) as a measurement for rating stability, so that the larger the CV, the larger the instability of a given size rating. Following this, we calculated the mean CV for each participant (across the different objects) and excluded size ratings with a CV higher than two standard deviations from the participant CV mean. Finally, we calculated the mean CV for each object (across different participants) and excluded objects with a CV higher than two standard deviations from the objects' CV mean (mean CV = .22, $SD = .065$).

This resulted in excluding 1 object: Basket (CV = 0.47). Mean conceptual size ratings are presented in Table S2.

Supplementary References

1. Paivio, A. Perceptual comparisons through the mind's eye. *Mem. Cogn.* **3**, 635-647 (1975).
2. Stevens, S. S. On the theory of scales of measurements. *Science* **103**, 677-680 (1946).
3. Rossion, B., & Pourtois, G. Revisiting Snodgrass and Vanderwart's object pictorial set: The role of surface detail in basic-level object recognition. *Perception* **33**, 217-236 (2004).

Table S1. The effect of physical size on speed of recognition

**Object**	***F***	***p*-value**
Basket	0.59	0.63
Bear	1.67	0.18
Bed	0.8	0.5
Bee	2.12	0.1
Bird	0.07	0.98
Book	0.43	0.73
Bus	1.07	0.37
Butterfly	0.13	0.94
Button	0.63	0.6
Camel	0.72	0.54
Cart	0.28	0.84
Cask	1.18	0.32
Chicken	1.05	0.38
Couch	0.38	0.77
Cow	0.47	0.71
Cup	0.58	0.63
Desk	1.96	0.13
Dog	0.7	0.55
Donkey	1.54	0.21
Door	0.25	0.86
Dresser	0.21	0.89
Duck	0.48	0.69
Elephant	0.12	0.95
Fish	0.92	0.44
Flower	0.83	0.48
Fly	2.4	0.075
Fox	1.6	0.2
Fridge	1.69	0.18
Frog	0.24	0.87
Garbage	0.54	0.66
Giraffe	1.38	0.26
Glass	0.61	0.61
Grapes	0.92	0.44
Hat	0.89	0.45
Horse	1.51	0.22
Leaf	0.32	0.81
Lemon	0.91	0.44
Leopard	1.08	0.36
Light	1.5	0.22
Motor	0.59	0.62
Mouse	1.85	0.15

Mushroom	0.06	0.98
Onion	0.2	0.9
Oven	0.73	0.54
Pear	0.85	0.47
Pen	0.91	0.44
Rabbit	1.77	0.16
Rhino	1.38	0.25
Snail	1	0.43
Strawberry	0.23	0.87
Table	0.35	0.79
Tea Pot	1.56	0.21
Toaster	1.15	0.33
Turtle	1.98	0.13
Zebra	0.67	0.57

Table S2. Mean conceptual size rating for each object.

Object	Size rating
Bear	3278
Bed	1583.82
Bee	6.21
Bird	27.6
Book	50.18
Butterfly	8.8
Button	4.55
Camel	3383.7
Cart	824.1
Cask	493.1
Chicken	80.97
Couch	1384.17
Cow	2053.7
Cup	27.2
Desk	1096.84
Dog	547.81
Donkey	1303.1
Door	1112.5
Dresser	969.26
Duck	81.99
Elephant	39522.73
Fish	31.67
Flower	17.31
Fox	511.2
Fridge	1667
Frog	26.49
Garbage	403.53
Giraffe	5572.4
Glass	24.8
Grapes	36.3
Hat	52.54
Horse	2327.71
Leaf	7.62
Lemon	24.8
Leopard	1260.83
Light	22.49
Motorcycle	1538.76
Mouse	25.23
Mushroom	11.15
Onion	24.98
Oven	1008.02

Pear	22.14
Pan	67.49
Rabbit	65.96
Rhino	5231.73
Snail	10.08
Strawberry	12.74
Table	1037.74
Tea Pot	66.94
Toaster	80.68
Turtle	61.21
Zebra	1346.74
